# WET: Word embedding-topic distribution vectors for MOOC video lectures dataset

**DOI:** 10.1016/j.dib.2019.105090

**Published:** 2020-01-03

**Authors:** Zenun Kastrati, Arianit Kurti, Ali Shariq Imran

**Affiliations:** a Dept. of Computer Science and Media Technology, Linnaeus University, Växjö, Sweden; b Dept. of Computer Science, Norwegian University of Science and Technology, Trondheim, Norway

**Keywords:** Word embedding, Document topics, Video lecture transcript, MOOC, LDA, Word2Vec

## Abstract

In this article, we present a dataset containing word embeddings and document topic distribution vectors generated from MOOCs video lecture transcripts. Transcripts of 12,032 video lectures from 200 courses were collected from Coursera learning platform. This large corpus of transcripts was used as input to two well-known NLP techniques, namely Word2Vec and Latent Dirichlet Allocation (LDA) to generate word embeddings and topic vectors, respectively. We used Word2Vec and LDA implementation in the Gensim package in Python. The data presented in this article are related to the research article entitled “Integrating word embeddings and document topics with deep learning in a video classification framework” [1]. The dataset is hosted in the Mendeley Data repository [2].

**Table:** Specifications Table

Subject	Computer Science
Specific subject area	Machine Learning, Natural Language Processing, Text Classification, eLearning
Type of data	Table in csv format
How data were acquired	Dataset was collected and created using video lectures and their corresponding transcripts gathered from a MOOC learning platform
Data format	Raw and Analyzed
Parameters for data collection	Standard text processing methods were applied
Description of data collection	The dataset contains word embeddings and document topics generated from MOOCs video lecture transcripts using Python's Gensim implementation of Word2Vec and LDA algorithms.
Data source location	Coursera MOOC learning platform
Data accessibility	Accessible on the Mendeley Data repository
Related research article	Zenun Kastrati, Ali Shariq Imran, and Arianit Kurti, Integrating word embeddings and document topics with deep learning in a video classification framework, Pattern Recognition Letters, 128C (2019) pp. 85–92, https://doi.org/10.1016/j.patrec.2019.08.019

**Table:** 

**Value of the Data** • This dataset is useful for the research community for two reasons: First, it is the first of its kind dataset aimed at video lectures classification using NLP techniques which is collected from the wild massive open online courses (MOOCs), and second, this dataset with its large-scale corpus size could serve as a standard benchmark for these research areas as well as for testing performance of the existing and new methods and techniques. • The research community in the fields of machine learning, information retrieval, video processing, education can benefit from these data by using them in various research tasks such as: transfer learning, video lectures classification and recommendation, contextual analysis, short text enrichment with topics, performance analysis of deep learning models and techniques, personalized learning. • Another possible value of these data is that they could be used and adopted from content providers and managers of MOOCs learning platforms to organize educational resources for maximum visibility and to easily search and find the best content.

## 1 Data

The key summary statistics of the MOOC video lecture transcripts corpus used to generate word embeddings and topic representation vectors is presented in Table 1. The dataset contains 12,032 video lecture transcripts that are composed of over 878 thousand sentences and more than 79 million tokens. The vocabulary size is over 68 thousand unique words.

**Table 1:** Statistics of the data used to generate word embeddings and topic representation vectors.

# of documents	# of sentences	# of tokens	vocabulary
12,032	878,209	79,680,144	68,176

**Fig. 1:**
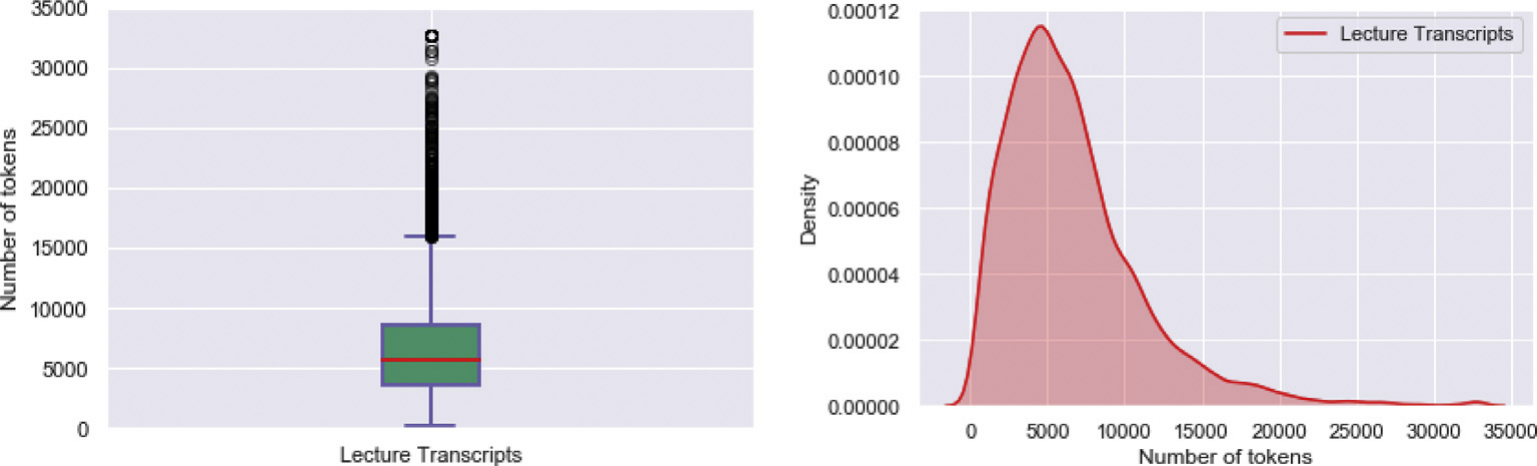
Length of video transcripts and distribution of tokens among them.

Video transcripts are of different length, from 228 to 32,767 tokens, with an average of 6622 tokens per video transcript. Video transcripts length variation illustrated in box plot and the distribution of tokens among the entire video transcripts corpus represented by a density function are shown in Fig. 1.

In Table 2, we show categories of the dataset including general-level and fined-grained along with the number of video lecture transcripts associated to each category.

**Table 2:** Distribution of video transcripts among general-level and fine-grained categories.

General-level Categories	Fine-grained Categories	# of docs
Art and Humanities	History	310
	Music and Art	338
	Philosophy	267
Physical Sciences and Engineering	Electrical Engineering	516
	Mechanical Engineering	287
	Chemistry	411
	Environmental Science and Sustainability	340
	Physics and Astronomy	455
	Research Methods	199
Computer Science	Software Development	284
	Mobile and Web Development	390
	Algorithms	338
	Computer Security and Networks	351
	Design and Product	228
Data Science	Data Analysis	205
	Machine Learning	549
	Probability and Statistics	283
Business	Leadership and Management	281
	Finance	346
	Marketing	242
	Entrepreneurship	216
	Business Essentials	223
	Business Strategy	261
Information Technology	Cloud Computing	171
	Security	139
	Data Management	236
	Networking	153
	Support and Operations	349
Health	Animal Health	227
	Basic Science	480
	Health Informatics	209
	Healthcare Management	167
	Patient Care	325
	Public Health	210
	Research	274
	Psychology	299
Social Sciences	Economics	516
	Education	293
	Governance and Society	331
	Law	333

**Fig. 2:**
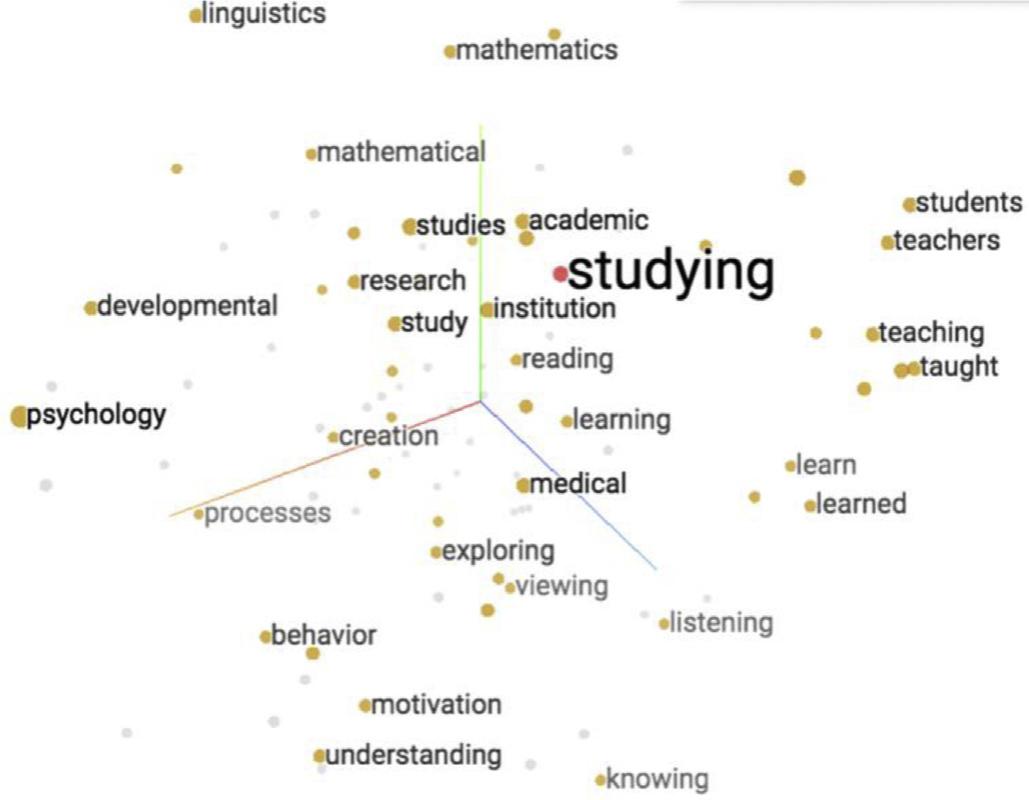
3D word embeddings visualization.

A visual representation of word embeddings generated from the MOOC video lectures corpora using principal component analysis (PCA) projected in a geometric space is illustrated in Fig. 2. More specifically, Fig. 2 shows an example of mapping of word ‘studying’ and its neighbours e.g. academic, studies, institution, reading, etc., in three-dimensional space.

## 2 Experimental design, materials, and methods

**Fig. 3:**
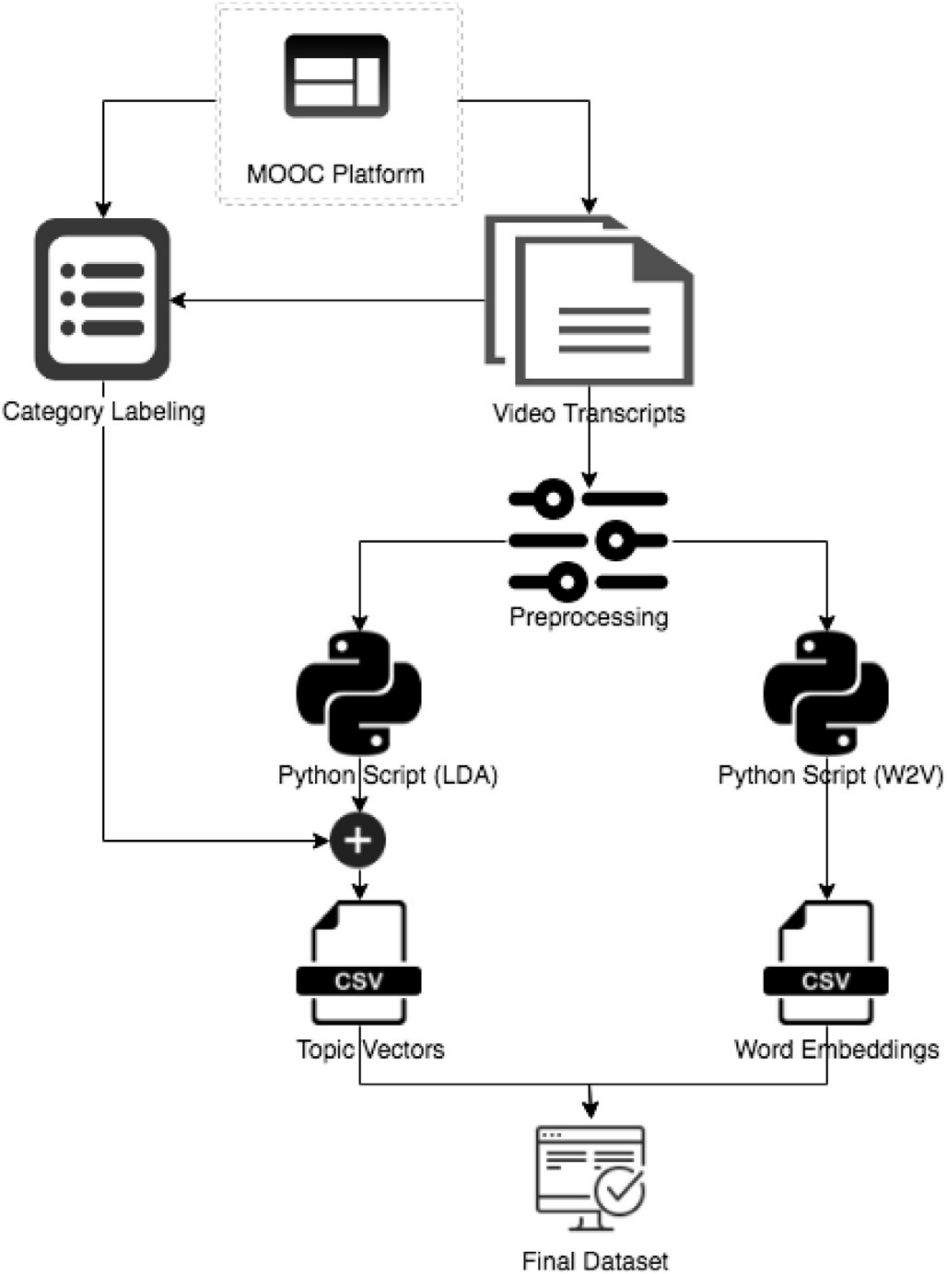
Dataset collection and creation scheme.

A new real-world dataset from the education domain is presented in this article. The dataset contains word embeddings and document topic distribution vectors generated by a corpus of 12,032 video lecture transcripts. The steps involved in collecting and creating the WET dataset are illustrated in Fig. 3.

As a first step, we downloaded video lecture transcripts from Coursera learning platform and annotated them. For annotation, we used a two-level hierarchical organizational structure of Coursera where each downloaded video transcript is associated with one fine-grained category and one general-level category of the structure. 8 general-level and 40 fine-grained categories constitute the dataset and the distribution of lecture transcripts among these categories is given in Table 2.

Prior to creating corpus and dictionary for generating word embeddings and topic distribution vectors, video lecture transcripts have undergone some preprocessing tasks including converting text to lowercase, removing stop words, punctuations, and removing words that are not purely comprised of alphabetical characters and those that are only one character. In addition, WordNetLemmatizer is used to lemmatize all words in transcripts. An open source Python Library for symbolic and statistical natural language processing called Natural Language Toolkit (NLTK) is used for performing preprocessing tasks.

### 2.1 Word embeddings

To train and generate word embeddings, we used the Word2Vec [3] word embedding technique implemented in Python's Gensim package [5]. Word2Vec is an unsupervised learning method in which word embeddings are learned using distribution of word co-occurrences within a local context, that is, a separate text window space scanned across the whole corpus. There are two model architecture of Word2Vec, namely the continuous bag-of-words (CBOW), and the skip-gram.

We set various parameters for Word2Vec as shown in Table 3. Word embeddings of different vector sizes including 50, 100, 200 and 300 dimensions, are generated.

**Table 3:** Parameters settings used for Word2Vec.

Parameter settings
*window size* = 5
*min_count* = 1
*alpha* = auto
*workers* = 4

Each of four csv files contains 68176 lines comprising of a unique word followed by either 50, 100, 200, or 300 real numbers that correspond to 50, 100, 200, 300 respective dimensions.

### 2.2 Topic distribution vectors

We conducted unsupervised topic modeling on the MOOC lecture transcript corpus. A latent Dirichlet allocation (LDA) [4] conventional topic modeling scheme implemented in the Python's Gensim package is used for generating document topic distribution vectors. LDA is a generative statistical model in which each document of a corpus is represented by a finite mixture of topics/themes which, in turn, are represented by a group of words. The parameter settings given in Table 4 are used for LDA and we performed training for varying number of topics including 50, 100, 200, 300 document topics.

**Table 4:** Parameters settings used for LDA.

Parameter settings
chunksize = 500
iterations = 400
passes = 20
alpha = auto
*eta* = auto

Each csv file contains 12,032 lines comprising of a unique word followed by either 50, 100, 200, or 300 real numbers that correspond to 50, 100, 200, 300 respective number of topics.

Concatenation of document topics obtained from LDA model with either general-level or fine-grained categories produces the final csv file as shown in Fig. 3.

## Conflict of Interest

The authors declare that they have no known competing financial interests or personal relationships that could have appeared to influence the work reported in this paper.
